# Supplementary material for: Disparities of women’s authorship in Colombia: A cross-sectional analysis
Source: PLOS Glob Public Health. 2025 Jan 22;5(1):e0003972. doi: 10.1371/journal.pgph.0003972 (PMC11753709; doi:10.1371/journal.pgph.0003972)
Supplement: Attachment — Submitted filename: Response to Reviewers V.4..docx [file pgph.0003972.s003.docx]

Zahra Zeinali, MD MPH DrGH (c)

Academic Editor, PLOS Global Public Health

August 29, 2024

**Response Letter for Manuscript PGPH-D-24-00677**

Dear Dr. Zeinali,

We are pleased to resubmit our manuscript titled *"* *Disparities of Women’s Authorship in Colombia: A Cross-Sectional Analysis "* to *PLOS Global Health.* We thank the Editors and the reviewers for their thoughtful reviews and helpful suggestions. We hereby respond point by point to each comment in both the response letter and in the revised manuscript.

The original reviewer comments are in bold, our response is in regular font, and the manuscript changes are tracked in the revised manuscript. We very much appreciate your continued interest in our manuscript and hope that you will consider the revised version for publication in *PLOS Global Public Health.*

Sincerely,

**María Alejandra Gutiérrez Torres, MD**

Postdoctoral Research Fellow

Yale Medicine | Department of Internal Medicine | Section of Cardiovascular Medicine

789 Howard Avenue, New Haven, CT 06519

Email: maria.a.gutierrez@yale.edu

**Comments to the Author**

**Reviewer 1:**

1. **You have used "women" while citing the literature, whereas you have used the term "female" throughout the manuscript. You might be aware the women is a more inclusive term and female refers to the biological sex at birth. Please justify the use of term "female" for your research or you actually mean women's under-representation in the context of their first authorship in impactful journals.**
   - Thank you very much for your insightful comment. We have revised the manuscript to replace all instances of 'female' with 'women' and 'male' with 'men'.
2. **You mention that "we performed an unadjusted logistic regression to explore the association between the first and last female authors". Please clarity because this sentence does not make it clear about what is the predictor and what are the explanatory variables. Explain the reason to use logistic regression. What hypothesis is the logistic regression testing? How many are research articles, how many are case reports and how many are review articles? Was it not possible to use one single logistic regression and use dummy variables for the type of article? Using dummy variables would give additional insights across these article categories.**
   - Following the author’s recommendations, we refined our statistical approach. The revised version reads as follows. The results of the multivariate models are shown below and are in the manuscript as Table 2 and Table 3, as well as in the results and discussion sections.

**Table 2.** Factors associated with the first author being a woman from 2018-2022 in Colombia.

| **Factors associated with the first author being a woman from 2018-2022 in Colombia** | | **Odds ratio** | **95% Confidence Interval** | **p-value** |
| --- | --- | --- | --- | --- |
| First author being women | | 1.55 | 1.39 - 1.73 | 0 |
| Subspeciality | Others | Reference | | |
|  | Cardiology | 0.63 | 0.49 - 0.80 | 0 |
|  | Gastroenterology | 0.34 | 0.26 - 0.45 | 0 |
|  | Anesthesiology | 0.67 | 0.46 - 0.98 | 0.036 |
|  | Dermatology | 2.44 | 1.62 - 3.68 | 0 |
|  | Infectiology | 1.15 | 0.86 - 1.54 | 0.339 |
|  | Neurology | 0.79 | 0.57 - 1.08 | 0.137 |
|  | Hematology | 0.79 | 0.56 - 1.12 | 0.191 |
|  | Public Health | 1.04 | 0.85 - 1.27 | 0.714 |
|  | Radiology | 1.47 | 0.97 - 2.22 | 0.071 |
|  | Sports Medicine and Rehabilitation | 0.64 | 0.44 - 0.93 | 0.02 |
|  | Pediatrics | 0.92 | 0.65 - 1.29 | 0.614 |
|  | Gynecology | 0.81 | 0.54 - 1.20 | 0.286 |
|  | Urology | 0.75 | 0.55 - 1.02 | 0.066 |
|  | Otorhinolaryngology (ENT) | 0.87 | 0.59 - 1.28 | 0.49 |
|  | Neurosurgery | 0.27 | 0.15 - 0.49 | 0 |
|  | Orthopedics / Traumatology | 0.1 | 0.06 - 0.18 | 0 |
|  | Psychiatry | 0.88 | 0.61 - 1.29 | 0.525 |
|  | Endocrinology | 0.62 | 0.45 - 0.86 | 0.004 |
|  | Nephrology | 0.47 | 0.31 - 0.72 | 0.001 |
|  | Critical Care | 0.56 | 0.40 - 0.78 | 0.001 |
|  | Surgery | 0.34 | 0.22 - 0.52 | 0 |
| Year | 2018 | Reference | | |
|  | 2019 | 1.07 | 0.90 - 1.27 | 0.461 |
|  | 2020 | 0.97 | 0.82 - 1.14 | 0.701 |
|  | 2021 | 0.95 | 0.80 - 1.11 | 0.499 |
|  | 2022 | 1.18 | 1.00 - 1.39 | 0.056 |
| Type of article | Original Research | Reference | | |
|  | Case Report | 0.73 | 0.64 - 0.84 | 0 |
|  | Review | 0.97 | 0.84 - 1.11 | 0.648 |
| City | Bogotá | Reference | | |
|  | Bucaramanga | 1.37 | 1.08 - 1.73 | 0.008 |
|  | Medellín | 1 | 0.86 - 1.17 | 0.975 |
|  | Cali | 0.87 | 0.70 - 1.07 | 0.187 |
|  | International | 0.77 | 0.60 - 0.98 | 0.033 |
|  | Other | 0.97 | 0.84 - 1.11 | 0.653 |

**Table 3.** Factors associated with the last author being a woman from 2018-2022 in Colombia.

| **Factors associated with the last author being a woman from 2018-2022 in Colombia** | | **Odds ratio** | **95% Confidence Interval** | **p-value** |
| --- | --- | --- | --- | --- |
| Last author being women | | 1.56 | 1.40 - 1.73 | 0 |
| Subspeciality | Others | Reference | | |
|  | Cardiology | 0.58 | 0.45 - 0.74 | 0 |
|  | Gastroenterology | 0.56 | 0.43 - 0.72 | 0 |
|  | Anesthesiology | 0.58 | 0.39 - 0.85 | 0.006 |
|  | Dermatology | 1.29 | 0.88 - 1.89 | 0.196 |
|  | Infectiology | 1.05 | 0.79 - 1.41 | 0.717 |
|  | Neurology | 0.83 | 0.60 - 1.14 | 0.251 |
|  | Hematology | 1.04 | 0.73 - 1.47 | 0.835 |
|  | Public Health | 0.99 | 0.81 - 1.21 | 0.927 |
|  | Radiology | 0.89 | 0.59 - 1.36 | 0.597 |
|  | Sports Medicine and Rehabilitation | 0.83 | 0.57 - 1.21 | 0.332 |
|  | Pediatrics | 1.34 | 0.96 - 1.88 | 0.087 |
|  | Gynecology | 0.68 | 0.45 - 1.03 | 0.068 |
|  | Urology | 0.5 | 0.35 - 0.70 | 0 |
|  | Otorhinolaryngology (ENT) | 0.89 | 0.60 - 1.31 | 0.547 |
|  | Neurosurgery | 0.29 | 0.16 - 0.54 | 0 |
|  | Orthopedics / Traumatology | 0.4 | 0.27 - 0.59 | 0 |
|  | Psychiatry | 0.89 | 0.61 - 1.30 | 0.548 |
|  | Endocrinology | 0.65 | 0.46 - 0.91 | 0.012 |
|  | Nephrology | 0.65 | 0.43 - 0.99 | 0.044 |
|  | Critical Care | 0.55 | 0.39 - 0.78 | 0.001 |
|  | Surgery | 0.57 | 0.38 - 0.87 | 0.008 |
| Year | 2018 | Reference | | |
|  | 2019 | 1 | 0.84 - 1.20 | 0.957 |
|  | 2020 | 1.12 | 0.94 - 1.32 | 0.197 |
|  | 2021 | 1.19 | 1.01 - 1.40 | 0.039 |
|  | 2022 | 0.97 | 0.82 - 1.15 | 0.697 |
| Type of article | Original Research | Reference | | |
|  | Case Report | 0.88 | 0.77 - 1.01 | 0.065 |
|  | Review | 1.11 | 0.97 - 1.27 | 0.14 |
| City | Bogotá | Reference | | |
|  | Bucaramanga | 0.96 | 0.76 - 1.21 | 0.732 |
|  | Medellín | 1.06 | 0.91 - 1.23 | 0.488 |
|  | Cali | 0.8 | 0.64 - 0.99 | 0.04 |
|  | International | 0.82 | 0.65 - 1.03 | 0.085 |
|  | Other | 1.05 | 0.91 - 1.21 | 0.51 |

**Reviewer 2:**

**Major comments:**

1. **While gender gap in STEM education is well known and a contemporary issue, how and why is looking at the proportion of papers with first female author a way to contextualize gender disparity in medical academia? I am making this point because:**
(a) **Many journals (and authors) in several disciplines now moved to ordering authors based on the alphabetic order of their names, which makes gender of the first author as random. Further, several journal in a few disciplines (say, economics) also publish a credit author statement – which reflects the contribution of each author irrespective of the order in which the name of the author(s) appear.**

**Since author(s) of this paper have partly motivated their research question based on the idea that the career progressions of females may be impacted by the first authorship of an individual, major comment 1(a) makes it less of a concern.**

- We value the feedback regarding the use of first authorship as a metric for understanding gender disparities in medical academia. In response, we have provided further explanation in our paper on the significance of this measure, particularly in the context of medical research. Unlike disciplines such as economics or physics, where author order may be alphabetical and not indicative of contribution, authorship in medical sciences is usually contribution-based. This is especially true in Colombia, where first authorship is a recognized metric in medical academia. Typically, the first author is the primary researcher, signifying significant involvement and impact on the project, which is essential for career advancement in this field. Understanding and acknowledging these disciplinary norms underscores the importance of first authorship in assessing gender disparities in academia. This insight is particularly relevant to our study's medical focus, justifying our methodological choice to highlight such inequalities.
  1.1 **Relatedly, why is last authorship an important outcome variable which the author(s) of this paper have looked upon in their analysis. If author(s) could also motivate why the female last author is an important outcome variable then it may help the reader to appreciate the findings of the paper more.**
- Thank you for your valuable feedback. We have expanded our discussion in the paper to explain better why last authorship is essential, especially in the context of academic research authorships. We detailed that the last authorship frequently indicates seniority and leadership within the research team, which are essential in project conception, supervision, and funding acquisition. Examining the representation of women in these positions allows us to investigate broader issues of gender equity in academic leadership and mentorship roles.

2. **I am not sure if the conclusion written in the Introduction section of the paper follows from the analysis undertaken in the paper.**

**More precisely, the author(s) state “*……..This will serve as the foundation for developing targeted interventions and policy recommendations to promote gender equity in the research ecosystem.***

***The anticipated results of this study are expected to stimulate collaborative efforts among policymakers, funding agencies, and research institutions to increase female representation and inclusivity in research throughout Latin American nations. By shedding light on the existing disparities and proposing evidence-based solutions, this study seeks to contribute to the creation of a fairer and more equitable academic environment in the region…..*.”**

**The claim that documenting of gender disparities can provide foundation for targeted interventions and create a fairer environment seems unlikely. Ideally, if the author(s) through an experiment, or, had undertaken an impact assessment of a policy (using quasi-experimental methods), had shown that certain policies can reduce gender-gaps -- then this conclusion would have followed.**

**However, in my opinion, the analysis of the paper is only suggestive of (or identifies) a stylized fact that females are less likely to be first authors of research articles and case studies in medical journals published in Colombia. They are not identifying any reasons for this problem or proposing any targeted intervention (that may work) for reducing this through their analysis.**

**Similarly, conclusions/claims in the discussion section may be revised by the author(s).**

- Thank you for your insightful comments. We have revised the text to emphasize that while our analysis highlights persistent gender disparities in first authorship among medical journals published in Colombia, it does not exhaustively identify specific causative factors or test targeted interventions. Instead, it identifies significant patterns intended to serve as a foundation for the future. We envision that our findings will spark discussions among policymakers, funding agencies, and research institutions about the critical need to investigate the root causes of gender inequity in academic authorship. Furthermore, we have stated that while our study does not propose specific interventions, it does identify important trends that merit serious consideration and further investigation.

**Minor points**

1. **Figure 3 that depicts heterogeneity in the proportion of females first-author based on sub-speciality indicates to me that there is a gender based self-selection in type of sub-speciality choses by individuals. For instance: In comparison to males, females may prefer more to be in sub-speciality jobs like nutrition/dermatology as compared to orthopedics/neurosurgery.**

**Thus, the lower proportion of females first author in orthopedics/neurosurgery journals may simply be due to lower number females in that sub-speciality (self-selection) and not gender disparity – which is typically perceived as gender discrimination.**

**Ideally for disparity/discrimination, one should do a relative analysis. For instance: out of the total females in nutrition/dermatology profession, how many females end up being as first authors of published papers. Similarly, out of the total males in nutrition/dermatology profession, how many females end up being as first authors of published papers. Comparison of these two figures can in true sense can serve to provide a sense of disparity in my opinion.**

- Thank you for observing the potential for gender-based self-selection into specific medical sub-specialties, which raises an important point. In response, we have adjusted our discussion to reflect better the complexities involved in accurately assessing gender disparities in authorship; also, in our regression, we controlled all the variables that we had available and added new tables (Table 2 and Table 3), nonetheless even if we tried to find the proportion of women in each sub-specialty those are numbers which are not available in the Colombian data which limits our findings, leading us to add this to the limitations section.

Recognizing the need for more detailed demographic data on the gender composition of each medical specialty in Colombia, we are advocating for the creation of a comprehensive database. This vital resource will enable us to precisely measure the proportion of women within each specialty, helping us determine whether disparities in authorship are influenced by systemic biases or simply by the distribution of women across different fields. This proactive step toward building a detailed database is fundamental for advancing our research and refining our interventions in the future since, by understanding the unique dynamics within each specialty, we can more effectively target our efforts to support women medical professionals, moving closer to achieving more accurate gender equity in authorship.

2. **I am unable to understand the Logistic regression (Table 2).**

**Ideally, the logistic regression should have covariates that may explain/predict the likelihood of a female first authorship in a paper. For instance: age of the author, type of journal, seniority of the researcher, number of males co-authors etc. This can serve to provide in true sense the determinants of female first authorship in a research paper. I suggest author(s) to undertake such analysis, if possible. In my opinion, this can add significant value to the paper.**

- Thank you for your comment. We have revised our logistic regression analysis and expanded it to include the variables we have available and have a revised description of the analysis with similar results. The statistical analysis now reads as follows:

The results now read as follows, as do Tables 2 and 3 showing the results of the model for women being first authors and women being last authors.

Unfortunately, due to most of the journals in Colombia not being indexed in major databases and requiring manual review of all the articles, some of the variables are not available at this time for analysis and will be part of future research efforts. Both limitations were added to the limitations section as follows:

Thirdly, our statistical analysis is limited due to the variables available in Colombia. Some variables, such as the number of authors and women enrolled in the different subspecialties, might also play a role and will be addressed in future research efforts.

**Response to Jay-ar Formentera Medes, editorial manager**

1. **Please provide separate figure files in .tif or .eps format and remove the embedded figures from the manuscript file.**
   - Thank you so much for your comment. It was corrected, and the figures are attached in .tif format.
2. **Please send a completed 'Competing Interests' statement, including any COIs declared by your co-authors. If you have no competing interests to declare, please state "The authors have declared that no competing interests exist".**
   - Thank you so much for your comment. It was corrected in the manuscript.
3. **Please amend your detailed Financial Disclosure statement. This is published with the article. It must therefore be completed in full sentences and contain the exact wording you wish to be published. If you did not receive any funding for this study, please simply state: “The authors received no specific funding for this work.”**
   - Thank you so much for your comment. It was corrected in the manuscript.
4. **Fig 4: please (a) provide a direct link to the base layer of the map (i.e., the country or region border shape) and ensure this is also included in the figure legend; and (b) provide a link to the terms of use / license information for the base layer image or shapefile. We cannot publish proprietary or copyrighted maps (e.g. Google Maps, Mapquest) and the terms of use for your map base layer must be compatible with our CC-BY 4.0 license. If you created the map in a software program like R or ArcGIS, please locate and indicate the source of the basemap shapefile onto which data has been plotted. If your map was obtained from a copyrighted source please amend the figure so that the base map used is from an openly available source. Alternatively, please provide explicit written permission from the copyright holder granting you the right to publish the material under our CC-BY 4.0 license.**
   - Thank you so much. We tried using the government website <https://www.colombiaenmapas.gov.co/> and [https://geoportal.igac.gov.co/contenido/datos-abiertos-cartografia-y-geografia,](https://geoportal.igac.gov.co/contenido/datos-abiertos-cartografia-y-geografia) but it wasn't easy to modify it based on what we wanted to represent. So, we used an online tool to redo the maps. Not before reading the terms of use in which they explicitly mentioned that any image created on the website could be used even for publication if we reference the website in the work. In our case, we used the image as it was downloaded with their reference in the right-down corner, and also in the text it is mentioned where we designed and the link to the website.

For you, to corroborate the information, here is the term of use URL: <https://www.mapchart.net/terms.html#licensing-maps>

Once again, thank you for the time.

5. **We notice that your supplementary [figures/tables] are included in the manuscript file. Please remove them and upload them with the file type 'Supporting Information'. Please ensure that each Supporting Information file has a legend listed in the manuscript before or after the references list.**
   - Thank you so much for your comment. It was corrected in the manuscript. Also, we are attaching a new file with the Supporting Information.
6. **We have noticed that you have uploaded Supporting Information files, but you have not included a list of legends. Please add a full list of legends for your Supporting Information files before or after the references list.**
   - Thank you so much for your comment. It was corrected in the manuscript, as well as in the Supporting Information file.
