# Supplementary material for: Disparities of women’s authorship in Colombia: A cross-sectional analysis
Source: PLOS Glob Public Health. 2025 Jan 22;5(1):e0003972. doi: 10.1371/journal.pgph.0003972 (PMC11753709; doi:10.1371/journal.pgph.0003972)
Supplement: Attachment — Submitted filename: Review_PGPH-D-24-00677R1.docx [file pgph.0003972.s004.docx]

I think the author(s) have thoroughly revised the paper and I appreciate their efforts. However, I just have 3 minor points to make:

1. Contribution to the literature: I still feel the author(s) state more than what the paper actually contributes. This is especially true when the author(s) write:

“*This study seeks to address these challenges by generating evidence-based recommendations for policy and practice, given the insufficient data regarding the scope and severity of gender inequality in scientific fields at the regional level.*”

I doubt if any evidence-based recommendations are generated for policy and practice. Rather, I feel it’s a stylized fact which the authors are highlighting about the gender inequality in Medical research.

2. Descriptive Statistics: Author(s) state that “*women’s first authorships increased slightly in 2019 and 2022 but decreased in 2021. Similarly, last women’s authorships increased in 2021 but decreased in 2019 and 2022, indicating a possible inverse relationship, as illustrated in* ***Figure 2***.”

To the naked eye, these differences are very small and it hard to conclude whether actually or not these point estimates are statistically different from one another. In order to make this claim concretely, we need confidence intervals attached to each year's mean value.

Logistic regression: While undertaking the logistic regression the author(s) state that “*When assessing factors associated with women being the last authors*, *the variables positively associated with this were the first author being a woman and the year of publication 2021*”

How can year of paper of publication be concluded as a determinant of gender disparity. Rather, the year of publication just represents the trend. Hence, in 2021, it was more likely that to have women as last authors compared to the base/reference year (2018). In my opinion, year of publication cannot be interpreted as a covariate determining the gender inequality.
