# Supplementary material for: Disparities of women’s authorship in Colombia: A cross-sectional analysis
Source: PLOS Glob Public Health. 2025 Jan 22;5(1):e0003972. doi: 10.1371/journal.pgph.0003972 (PMC11753709; doi:10.1371/journal.pgph.0003972)
Supplement: Attachment — Submitted filename: Response to Reviewers 10.14.2024.docx [file pgph.0003972.s005.docx]

Zahra Zeinali, MD MPH DrGH (c)

Academic Editor, PLOS Global Public Health

November 2, 2024

**Response Letter for Manuscript PGPH-D-24-00677**

Dear Dr. Zeinali,

We are pleased to resubmit our manuscript titled *"* *Disparities of Women’s Authorship in Colombia: A Cross-Sectional Analysis "* to *PLOS Global Health.* We thank the Editors and the reviewers for their thoughtful reviews and helpful suggestions. We hereby respond point by point to each comment in both the response letter and in the revised manuscript.

The original reviewer comments are in bold, our response is in regular font, and the manuscript changes are tracked in the revised manuscript. We very much appreciate your continued interest in our manuscript and hope that you will consider the revised version for publication in *PLOS Global Public Health.*

Sincerely,

**María Alejandra Gutiérrez Torres, MD**

Postdoctoral Research Fellow

Yale Medicine | Yale Child Study Center

230 S Frontage Rd | New Haven, CT 06519

Email: maria.a.gutierrez@yale.edu

**Comments to the Author**

**Reviewer 1:**

1. **I still feel the author(s) state more than what the paper actually contributes. This is especially true when the author(s) write: “*This study seeks to address these challenges by generating evidence-based recommendations for policy and practice, given the insufficient data regarding the scope and severity of gender inequality in scientific fields at the regional level.*” I doubt if any evidence-based recommendations are generated for policy and practice. Rather, I feel it’s a stylized fact which the authors are highlighting about the gender inequality in Medical research.**

- Thank you for your feedback. We now clearly delineate between the foundational facts we've highlighted about gender inequality in medical research and the potential pathways for future evidence-based recommendations. This change clarifies our actual contributions and sets a more accurate expectation for the study's scope. We appreciate your input and believe this revision improves the clarity of our study.

2. **Author(s) state that “*women’s first authorships increased slightly in 2019 and 2022 but decreased in 2021. Similarly, last women’s authorships increased in 2021 but decreased in 2019 and 2022, indicating a possible inverse relationship, as illustrated in Figure 2*.” To the naked eye, these differences are very small and it hard to conclude whether actually or not these point estimates are statistically different from one another. In order to make this claim concretely, we need confidence intervals attached to each year's mean value. While undertaking the logistic regression the author(s) state that “*When assessing factors associated with women being the last authors*, *the variables positively associated with this were the first author being a woman and the year of publication 2021*”. How can year of paper of publication be concluded as a determinant of gender disparity. Rather, the year of publication just represents the trend. Hence, in 2021, it was more likely that to have women as last authors compared to the base/reference year (2018). In my opinion, year of publication cannot be interpreted as a covariate determining the gender inequality.**

- Thank you for your comment. Taking your comment into account, we have changed the way we present the year of publication. Now, having rewritten the necessary parts of the analysis, we illustrate a trend rather than imply causality, as you state. We have revised the text to clarify that this variable is intended to represent a trend in gender authorships over time. We have made adjustments to ensure there is no confusion regarding the interpretation of this variable.
