# Supplementary material for: Disparities of women’s authorship in Colombia: A cross-sectional analysis
Source: PLOS Glob Public Health. 2025 Jan 22;5(1):e0003972. doi: 10.1371/journal.pgph.0003972 (PMC11753709; doi:10.1371/journal.pgph.0003972)
Supplement: Attachment — Submitted filename: Referee report.docx [file pgph.0003972.s002.docx]

Referee report on “**Disparities of Female Authorship in Colombia: A Cross-Sectional Analysis**”

The paper attempts to document underrepresentation of females as first (and last) author(s) of research articles and case studies in medical journals published in Colombia. The author(s) shortlisted journals and articles published in Colombian jounrals from the year 2018 till 2022. They find that females are less likely to be first (and last) author(s) with considerable heterogeneity across journals depending upon the journal’s sub-speciality.

The research question is really intriguing and worth exploring. I have a handful of comments for the author(s) to think upon.

Major comments:

1. While gender gap in STEM education is well known and a contemporary issue, how and why is looking at the proportion of papers with first female author a way to contextualize gender disparity in medical academia? I am making this point because:
(a) Many journals (and authors) in several disciplines now moved to ordering authors based on the alphabetic order of their names, which makes gender of the first author as random. Further, several journal in a few disciplines (say, economics) also publish a credit author statement – which reflects the contribution of each author irrespective of the order in which the name of the author(s) appear.

Since author(s) of this paper have partly motivated their research question based on the idea that the career progressions of females may be impacted by the first authorship of an individual, major comment 1(a) makes it less of a concern.

1.1 Relatedly, why is last authorship an important outcome variable which the author(s) of this paper have looked upon in their analysis. If author(s) could also motivate why the female last author is an important outcome variable then it may help the reader to appreciate the findings of the paper more.

2. I am not sure if the conclusion written in the Introduction section of the paper follows from the analysis undertaken in the paper.

More precisely, the author(s) state “*……..This will serve as the foundation for developing targeted interventions and policy recommendations to promote gender equity in the research ecosystem.*

*The anticipated results of this study are expected to stimulate collaborative efforts among policymakers, funding agencies, and research institutions to increase female representation and inclusivity in research throughout Latin American nations. By shedding light on the existing disparities and proposing evidence-based solutions, this study seeks to contribute to the creation of a fairer and more equitable academic environment in the region…..*.”

The claim that documenting of gender disparities can provide foundation for targeted interventions and create a fairer environment seems unlikely. Ideally, if the author(s) through an experiment, or, had undertaken an impact assessment of a policy (using quasi-experimental methods), had shown that certain policies can reduce gender-gaps -- then this conclusion would have followed.

However, in my opinion, the analysis of the paper is only suggestive of (or identifies) a stylized fact that females are less likely to be first authors of research articles and case studies in medical journals published in Colombia. They are not identifying any reasons for this problem or proposing any targeted intervention (that may work) for reducing this through their analysis.

Similarly, conclusions/claims in the discussion section may be revised by the author(s).

Minor points

1. Figure 3 that depicts heterogeneity in the proportion of females first-author based on sub-speciality indicates to me that there is a gender based self-selection in type of sub-speciality choses by individuals. For instance: In comparison to males, females may prefer more to be in sub-speciality jobs like nutrition/dermatology as compared to orthopedics/neurosurgery.

Thus, the lower proportion of females first author in orthopedics/neurosurgery journals may simply be due to lower number females in that sub-speciality (self-selection) and not gender disparity – which is typically perceived as gender discrimination.

Ideally for disparity/discrimination, one should do a relative analysis. For instance: out of the total females in nutrition/dermatology profession, how many females end up being as first authors of published papers. Similarly, out of the total males in nutrition/dermatology profession, how many females end up being as first authors of published papers. Comparison of these two figures can in true sense can serve to provide a sense of disparity in my opinion.

2. I am unable to understand the Logistic regression (Table 2).

Ideally, the logistic regression should have covariates that may explain/predict the likelihood of a female first authorship in a paper. For instance: age of the author, type of journal, seniority of the researcher, number of males co-authors etc. This can serve to provide in true sense the determinants of female first authorship in a research paper. I suggest author(s) to undertake such analysis, if possible. In my opinion, this can add significant value to the paper.
